# Serum concentrations of haptoglobin and serum amyloid A in water buffaloes (*Bubalus bubalis*) with abomasal ulcer

**Published:** 2012

**Authors:** Javad Tajik, Saeed Nazifi, Mahdi Heidari, Marzieh Babazadeh

**Affiliations:** 1 *Department of Clinical Studies, School of Veterinary Medicine, Shahid Bahonar University of Kerman, Kerman, Iran; *; 2 *Department of Clinical Studies, School of Veterinary Medicine; Shiraz University, Shiraz, Iran*

**Keywords:** Haptoglobin, Serum amyloid A, Abomasal ulcer, * Bubalus bubalis*

## Abstract

To evaluate the serum concentrations of haptoglobin (Hp) and serum amyloid A (SAA) in water buffaloes with abomasal ulcers, the abomasums of 100 randomly selected water buffaloes were examined after slaughter. Type I abomasal ulcers were found in 56 out of 100 buffaloes. Serum concentrations of Hp and SAA were measured. There was no significant difference between affected and non-affected buffaloes in the serum concentrations of Hp and SAA. The serum concentrations of Hp and SAA had no significant correlation with age and the serum SAA revealed no significant correlation with the number of abomasal ulcers. A significant correlation was found between the serum Hp and the number of abomasal ulcers (r =0.29, *p* = 0.04). There was no significant difference in the serum concentrations of Hp and SAA between buffaloes with different ulcer locations in the abomasums. Although more work on a larger number of animals is required in this area, it seems that the measurement of the serum Hp can be used to predict the abundance of type I abomasal ulcers.

## Introduction

There are numerous reports regarding the occurrence of gastric ulcers in human, cattle, and swine. Different age groups of ruminants may be affected by abomasal ulcers.^1^ Abomasal ulcers were divided into four types, each type producing distinct clinical signs. However, Type I ulcers, non-perforating erosions of abomasal mucosa, have mild or undetectable clinical signs and are usually not diagnosed until slaughter.^2^ Braun *et al*. reported that 20.5 % of slaughtered cows had type I abomasal ulcer.^1^ The results of Aukema and Breukink's study showed that 6.3 % of slaughtered cows had abomasal ulcers or ulcer scars.^3^ Although the etiology and pathogenesis of abomasal ulcers are not completely known, the clinical status of animals, nutrition, concurrent diseases and stress factors have been proposed as the probable factors causing the different prevalence rates of abomasal ulcers reported in different studies.^1^^,^^4^^,^^5^ Braun *et al*. believe that clinically normal animals have mainly, type I ulcers, whereas animals with clinical signs of disease often have type 2 to 4 ulcers.^1^ Although the economic effect of abomasal erosions in ruminants has not yet been investigated, physical discomfort resulting in reduced feed consumption and weight gain have been proposed as the probable causes of loss.^6^

Iranian water buffalo (*Bubalus bubalis*) has a high economic value by providing meat, milk and labor for local farmers. Ghadrdan-Mashhadi *et al*. reported a high prevalence of type I abomasal ulcers (63.5 %) in slaughtered water buffaloes in Iran.^7^ Despite the probable importance of abomasal ulcers in water buffalo in Iran, there is a little information about the clinical signs and no previous study regarding the diagnosis of the abomasal ulcers in this species has been recorded.

Acute phase proteins (APPs) are a group of blood proteins that show changes in their concentration in animals subjected to external or internal challenges such as infection, inflammation, surgical trauma, or stress.^8^ It has been suggested that APPs may provide valuable diagnostic information in detection, prognosis and monitoring the diseases in animal species and animal health.^9^

Despite the growing interest in using APPs as health indicators,^10^ to the best of our knowledge, there has been no previous study regarding the probable changes of the APPs in abomasal ulcers in water buffalo. Therefore, the aim of this study was to evaluate and to compare the rate of serum haptoglobin (Hp) and serum amyloid A (SAA) between affected and non-affected water buffalo.

## Materials and Methods

**Animals and sampling. **The investigation was carried out on water buffaloes (*Bulbalus bubalis*) which were slaughtered in a slaughter house reserved only for buffaloes in Ahvaz City, southwestern Iran, from December 2009 to January 2010, and April to May 2010.

After clinical examination and before slaughter, jugular blood samples were collected from 100 clinically healthy water buffaloes in plane tubes, without anticoagulant. Immediately after slaughter, the abomasum of the animals were opened along the greater curvature and washed in water to free ingesta. The abomasal mucosa was examined, and the type, number, and location of the lesions were recorded. The most frequent type of lesion was referred to as the main lesion.^1^ Buffaloes were of both sexes with different ages, and were selected randomly. The age of the animals was estimated using dental characteristics. All animals had grazed the previous summer on ranges around the city. The blood serum was separated after centrifugation at 1800 *g *for 10 min and the serum samples stored at -20 ˚C until analysis.

**Assays**. Total serum protein (TP) was measured by the Biuret method and serum concentration of Hp was measured according to prevention of the peroxidase activity of hemoglobin, which is directly proportional to the amount of Hp.^10^ The analytical sensitivity of this test in serum has been determined as 0.01 mg mL^-1^ for Hp by the manufacturer (Tridelta Development Plc, Wicklow, Ireland).

Serum SAA was measured by a solid phase sandwich ELISA.^10^ The analytical sensitivity of this test in serum has been determined as 0.30 µg mL^-1^ for SAA by the manufacturer (Tridelta Development Plc, Wicklow, Ireland). 

**Statistical analysis. **Statistical analysis was performed using SPSS version 12 (Illinois, Chicago). The correlation of the measured serum parameters with age were analyzed by Pearson’s correlation tests. Two sample *t*-tests were used to detect differences in the parameters between the two sexes and between affected and non-affected buffaloes. The buffaloes were divided into three groups, according to their age as G_1_ ≤ 2 years, 2 years < G_2_ ≤ 5 years, and G_3_ > 5 years. Analysis of variance (ANOVA) tests were used to compare the serum proteins between the different age groups of water buffaloes. Differences were considered significant at *p* < 0.05.

## Results

Overall, 69 male buffaloes and 31 female buffaloes were sampled. The average ages (Mean ± SEM) of the male and female buffaloes were 2.19 ± 0.10 and 2.68 ± 0.30 years, respectively. There was no significant difference between the two sexes in age. 

The results of the serum concentration of measured proteins in affected and non-affected buffaloes and in both sexes are shown in Table 1. 

Fifty buffaloes were examined in each sampling period and 43 and 20 buffaloes were diagnosed as affected in the first and second sampling periods, respectively.

All abomasal lesions were classified as type I abomasal ulcers, and were confirmed in histopathological examinations.

There was no significant difference between affected and non-affected buffaloes in the serum concentrations of Hp and SAA (*p* > 0.05). There was also no significant difference between the affected and non-affected buffaloes in age (2.45 ± 0.16 and 2.07 ± 0.16, respectively) (*p* > 0.05). The serum concentrations of Hp and SAA had no significant correlation with age and the serum SAA revealed no significant correlation with the number of abomasal ulcers, however, serum Hp showed a significant correlation with the number of abomasal ulcers (r=0.29, *p* =0.04). 

There was no significant difference in the serum concentrations of Hp and SAA between buffaloes with different ulcer locations in the abomasums and between different age groups (*p* > 0.05).

**Table 1:** The concentrations (Mean ± SEM) of serum Hp and SAA in affected and non-affected buffaloes and in different genders and age groups

**Groups**		**No.**	**TP (g dL** ^-1^ **)**			**Hp (g L** ^-1^ **)**	**SAA (μg mL** ^-1^ **)**	
**All sampled**		100	7.16 ± 0.06			0.57 ± 0.06	4.11 ± 0.38	
**Affected**		63	7.08 ±0.07			0.56 ± 0.06	4.27 ± 0.44	
**Non-affected**		37	7.32 ±0.10			0.62 ± 0.19	3.18 ± 0.45	
**Male**		69	7.15 ± 0.07			0.55 ± 0.07	4.17 ± 0.54	
**Female**		31	7.18 ±0.12			0.61 ± 0.09	4.03 ± 0.49	
**G**_1_	64			7.21 ± 0.08	0.53 ± 0.07			4.16 ± 0.46	
**G**_2_	33			7.09 ±0.10	0.71 ± 0.14			4.27 ± 0.81	
**G**_3_	3			6.98 ± 0.29	0.35 ± 0.12			2.84 ± 0.79	

Both sexes were evaluated separately, and the results of the comparison of the measured serum factors between the affected and non-affected buffaloes showed no difference (*p* > 0.05).

Different age groups of buffaloes were also evaluated separately and the results of the comparison of the measured serum factors between the affected and non-affected buffaloes showed no difference (*p* > 0.05).

## Discussion

The reference value of serum Hp and SAA in healthy water buffaloes has been investigated and the results of Ghadrdan-Mashhadi *et al. *and the current studies have revealed the high prevalence of type I abomasal ulcers in water buffaloes in Iran.^7^^,^^11^ However, to the best of our knowledge, there has been no previous research regarding the changes of the serum Hp and SAA in abomasal ulcers in water buffaloes. The mean serum concentration of Hp for healthy water buffaloes in the current study (Mean ± SEM: 0.62 ± 0.19) was slightly higher than that previously reported as the normal ranges for healthy water buffaloes (0.13 - 0.06 g L^-1^) by Tajik et al.^11^ According to our results, the mean serum concentration of SAA for healthy water buffaloes was 3.18 ± 0.45 µg mL^-1^ and was slightly higher than the previously reported mean serum concentration of SAA in healthy water buffaloes (2.99 ± 0.14 μg mL^-1^).^11^ Some factors such as age, sex, breed, season, geographic and dietary factors affect the serum proteins,^12^^,^^13^ which may cause the observed differences.

Following tissue destruction and inflammation, change in blood serum proteins is expected.^14^ During inflammation, the protein production in liver is switched towards increased synthesis of positive acute phase proteins such as Hp and SAA.^8^^,^^14^^,^^15^ Haptoglobin is synthesized in the liver and is a major acute-phase protein in numerous species of animals.^16^^,^^17^ It is believed that the circulating level of Hp in ruminants is negligible in normal animals, however, increases over 100-fold with immune stimulation.^18^^,^^19^ Serum amyloid A is also considered as one of the major acute phase reactants in vertebrate,^10^ and it is generally assumed that SAA have a protective role during inflammation.^20^ According to our results, although there was no significant difference between affected and non-affected buffaloes in the serum concentrations of Hp and SAA, there was a significant correlation between the serum Hp and the number of abomasal ulcers. All detected abomasal ulcers in examined buffaloes were classified as type I, non-perforating erosions of abomasal mucosa, which are mucosal defects and do not penetrate the deeper layers of abomasum.^5^ It seems that little penetration of type I abomasal ulcer to the abomasal layers with no consequences, such as peritonitis (which occurs in type III and IV abomasal ulcer) or bleeding (which occurs in type II abomasal ulcer), causes a negligible effect on the blood serum Hp and SAA concentrations. However, the observed positive correlation between the number of abomasal ulcers and the serum Hp may be due to the moderate inflammatory reactions at the ulcer places, which were confirmed in the histopathological examination.

We found no significant difference between either sex in the serum concentrations of the measured acute-phase proteins, which was the same as that reported previously in water buffalo and other species, such as cattle and sheep.^11^^,^^21^^,^^22^

Our results also showed that serum Hp and SAA had no significant correlation with age and there was no significant difference between different age groups. Similar results have been found in previous studies in water buffalo, sheep and cattle.^11^^,^^21^^,^^22^ However, age-related changes in serum concentrations of Hp and SAA have been reported in horse.^23^^-^^25^ It is believed that species variations in APPs occur due to inherent variations in synthesis and in rates of entry and exit of these APPs from circulation.^26^

It has been shown that the occurrence and severity of inflammatory diseases in different species of domestic animals can be evaluated by acute phase protein profiling.^10^^,^^17^^,^^27^ It is believed that the stage of disease can be better evaluated by monitoring more than one acute phase protein.^15^ The findings of the present study showed that although measurement of serum Hp and SAA cannot be used solely in the diagnosis of type I abomasal ulcers in water buffaloes, measurement of the serum Hp can be used in prediction of the abundance of the type I abomasal ulcers. Different affected abomasal layers and distinctive consequences of the different types of abomasal ulcers such as local peritonitis in type III abomasal ulcers cause their effects on the serum proteins to be different and incomparable. It seems that more work on a larger number of animals and in other types of abomasal ulcers is needed before these findings can be used in practice.
